# Development and evaluation of machine learning models for voxel dose predictions in online adaptive magnetic resonance guided radiation therapy

**DOI:** 10.1002/acm2.12884

**Published:** 2020-04-19

**Authors:** M. Allan Thomas, Yabo Fu, Deshan Yang

**Affiliations:** ^1^ Department of Radiation Oncology Washington University in St. Louis St. Louis MO USA; ^2^ Present address: Department of Imaging Physics UT MD Anderson Cancer Center Houston TX USA; ^3^ Present address: Department of Radiation Oncology Emory University School of Medicine Atlanta GA USA

**Keywords:** adaptive radiation therapy, dose prediction, knowledge‐based prediction, magnetic resonance image guidance

## Abstract

**Purpose:**

Daily online adaptive plan quality in magnetic resonance imaging guided radiation therapy (MRgRT) is difficult to assess in relation to the fully optimized, high quality plans traditionally established offline. Machine learning prediction models developed in this work are capable of predicting 3D dose distributions, enabling the evaluation of online adaptive plan quality to better inform adaptive decision‐making in MRgRT.

**Methods:**

Artificial neural networks predicted 3D dose distributions from input variables related to patient anatomy, geometry, and target/organ‐at‐risk relationships in over 300 treatment plans from 53 patients receiving adaptive, linac‐based MRgRT for abdominal cancers. The models do not include any beam related variables such as beam angles or fluence and were optimized to balance errors related to raw dose and specific plan quality metrics used to guide daily online adaptive decisions.

**Results:**

Averaged over all plans, the dose prediction error and the absolute error were 0.1 ± 3.4 Gy (0.1 ± 6.2%) and 3.5 ± 2.4 Gy (6.4 ± 4.3%) respectively. Plan metric prediction errors were −0.1 ± 1.5%, −0.5 ± 2.1%, −0.9 ± 2.2 Gy, and 0.1 ± 2.7 Gy for V95, V100, D95, and D_mean_ respectively. Plan metric prediction absolute errors were 1.1 ± 1.1%, 1.5 ± 1.5%, 1.9 ± 1.4 Gy, and 2.2 ± 1.6 Gy. Approximately 10% (25) of the plans studied were clearly identified by the prediction models as inferior quality plans needing further optimization and refinement.

**Conclusion:**

Machine learning prediction models for treatment plan 3D dose distributions in online adaptive MRgRT were developed and tested. Clinical integration of the models requires minimal effort, producing 3D dose predictions for a new patient’s plan using only target and OAR structures as inputs. These models can enable improved workflows for MRgRT through more informed plan optimization and plan quality assessment in real time.

## 1 INTRODUCTION

The efficacy of radiation therapy (RT) for abdominal cancers is limited by a prescription dose (Rx) that can be tolerated without toxicity in nearby organs‐at‐risk (OARs). The abdominal OARs (liver, kidney, and all GI organs, etc.) are highly deformable in nature and their daily motion throughout treatment is substantial. Interfraction motion over 3 cm is possible.^1^, ^2^, ^3^ GI OARs (stomach, duodenum, esophagus, small and large intestines) are also sensitive to high doses of radiation.^4^, ^5^ As a result, tumors associated with such difficult abdominal sites have not been treated to the same curative doses that have been achieved for other sites, ie a biologically effective dose (BED) >100 Gy.^6^, ^7^, ^8^ Daily, online adaptive treatment with magnetic resonance image guided RT (MRgRT) can provide an opportunity to simultaneously escalate gross tumor volume (GTV) dose while meeting OAR constraints in these difficult cases.^9^ Both modeling studies^3^, ^10^, ^11^ and completed phase I clinical trials^12^, ^13^, ^14^ have shown the feasability of ablative dose escalation without significant adjacent organ toxicity in the central thorax and abdomen. Such trials have shown an increase in both local control and overall survival while decreasing radiation‐induced toxicity events, leading to a prospective clinical trial for further evaluation in pancreatic cancer (SMART: NCT03621644).

As the use of online adaptive MRgRT has grown recently across a variety of clinics, both the potential benefits and practical difficulties of its utilization are beginning to be understood.^12^, ^13^, ^14^, ^15^, ^16^, ^17^, ^18^, ^19^, ^20^, ^21^ But ample opportunity remains for assessment and improvement of online adaptive RT as this unique treatment method continues to develop clinically. Analysis of a collection of patient data and their treatment plans in order to better understand and improve a particular RT process can proceed in a variety of ways. But the most common method used recently falls under the general term of knowledge‐based (KB) prediction models.^22^ KB methods have been used to predict achievable dose‐volume histograms (DVHs),^23^, ^24^ plan metrics,^25^ and full 3D dose distributions.^26^, ^27^, ^28^, ^29^, ^30^, ^31^ In fact, using information from previously treated patients and plans to better inform future procedures is now present in nearly all aspects of RT.^32^, ^33^, ^34^, ^35^ Much of the recent work related to KB methods for improving RT has focused on 3D dose prediction: voxel‐by‐voxel prediction of dose for a “future” patient based on a collection of previously treated patients’ plans and/or other relevant data. Such a focus is understandable because the ability to accurately and robustly predict 3D dose volumes for future patients is significant. It produces in a single model the ability to predict a wide variety of clinically relevant data such as target and OAR dose‐volume metrics as well as conformity/homogeneity indices which may also be important. Furthermore, 3D dose predictions could serve as novel inputs for treatment plan optimization that may completely alter the paradigm under which RT planning currently occurs.^26^, ^28^, ^30^

Despite the growth in applications of KB methods to solving interesting RT problems, a majority of the most relevant work has focused on treatment sites such as prostate,^23^, ^24^, ^26^, ^28^, ^31^ head and neck,^25^, ^29^, ^30^ breast,^28^ and lung.^36^ There are limited studies dedicated to abdominal cancers^15^, ^27^ because they are notoriously difficult to treat and there is minimal consensus regarding the best RT treatment approaches.^37^ These sites are however expected to benefit most directly from MRgRT and online plan adaptation. Application of KB methods to online adaptive RT is also new, with very few previous studies.^15^ KB methods typically require sufficient data collection from previously treated patients and plans, and online adaptive RT is a mostly new technique.

In this work, we have developed artificial neural network (ANN) models to predict 3D dose distributions within the GTV for MRgRT of abdominal cancers. The clinical goals are to enable real‐time guidance for achievable plan quality of each online adaptive case, identify inferior plans, and assure plan quality. Unlike some previous KB works,^26^, ^27^ our ANN models do not require beam related variables such as beam angles and fluence. They therefore can predict a 3D dose distribution for a new patient’s plan using only target and OAR structures as inputs. In addition, the ANN models are simple in design and rely on small datasets. They do not require significant training time and are not computationally demanding (no GPU required). As a result, they can be integrated clinically with minimal effort. To our knowledge, this work presents the development and results of the first 3D dose prediction models for online adaptive MRgRT.

## 2 MATERIALS AND METHODS

### 2.A Patient Characteristics

As shown in Table 1, datasets from a total of 53 patients with abdominal cancers treated at Washington University in St. Louis with online adaptive, linac‐based MRgRT were utilized. The patient data were combined from two main treatment site groups, pancreas (n = 34) and nonpancreas (n = 19). All patients were treated with one of two high BED protocols: stereotactic body RT (SBRT, n = 48), having prescriptions of 50 Gy in 5 fractions (BED ~ 100 Gy) and critical OAR (OAR_CRIT_) dose constraints of less than 0.5 cubic centimeters (cc) receiving 36 Gy or more (V36 < 0.5 cc); or hypofractionation (HYP, n = 5) having prescriptions of 67.5 Gy in 15 fractions (BED ~ 100 Gy) and OAR_CRIT_ dose constraints of V50 < 0.5 cc. Overall, 260 out of 315 (82.5%) treatment fractions were adapted.

**Table 1:** Statistics for the plans and GTV V95% and D95% values.

Patient Group	Type of Plan	# of Plans			V95%	D95%
		SBRT	HYP	Total	Mean ± σ	Mean ± σ
Pancreas	Simulation^a^	29	4	33	86.2 ± 8.7	80.7 ± 12.6
	Adapted	133	56	189	86.2 ± 8.8	78.5 ± 15.4
NonPancreas	Simulation	16	1	17	94.6 ± 11.0	98.9 ± 13.0
	Adapted	56	15	71	92.5 ± 10.3	94.1 ± 14.5
All Patients	Simulation	45	5	50	89.3 ± 10.4	87.5 ± 15.5
	Adapted	189	71	260	87.9 ± 9.6	82.7 ± 16.6

^a^ Simulation plans are created on the patient's simulation image, receive normal planning time, attention, optimization, and checks just like traditional IMRT plans, and serve as the starting point of plan adaptation for the first fraction. V95% is the percentage of the GTV volume receiving 95% or more of Rx. D95% is the minimum dose received by 95% of the GTV, given as a percentage of Rx.

### 2.B Treatment Plan Adaptation Techniques

Detailed descriptions of the specific workflows and planning strategies for adaptive MRgRT using the ViewRay linac system (Viewray, Cleveland, OH) have been presented previously.^12^, ^13^, ^21^, ^38^, ^39^, ^40^, ^41^ A brief summary, with particular focus on the details related to the online adaptive process, follows.

Table 2 includes key definitions for structures associated with the planning process. Additional OARs beyond those in OAR_CRIT_ may have been included in the plan optimization such as the spinal cord, liver, aorta, and one or both kidneys. PTV_OPT_ is used as the target in the plan optimization instead of the PTV. It is often observed in these plans that, after subtracting OAR_5 mm_, not all of the GTV will be inside PTV_OPT_. The planning strategy is quite different from traditional IMRT. PTV V95>=95% was still one planning goal, but the hard OAR_CRIT_ dose constraints caused this target coverage goal to be rarely achievable. Hotspots were generally limited to <150% of the Rx inside the GTV.

**Table 2:** Structure names and definitions used throughout this work.

**Planning Structure**	**Definition**
GTV	Gross tumor volume
PTV	GTV + 5 mm isotropic expansion
OAR_CRIT_	All abdomen organs in close proximity of GTV (esophagus, stomach, duodenum, large bowel, small bowel)
OAR_5mm_	OAR_CRIT_ + 5 mm isotropic expansion
PTV_OPT_	PTV minus OAR_5 mm_: used as target in plan optimization

**Fig. 1:**
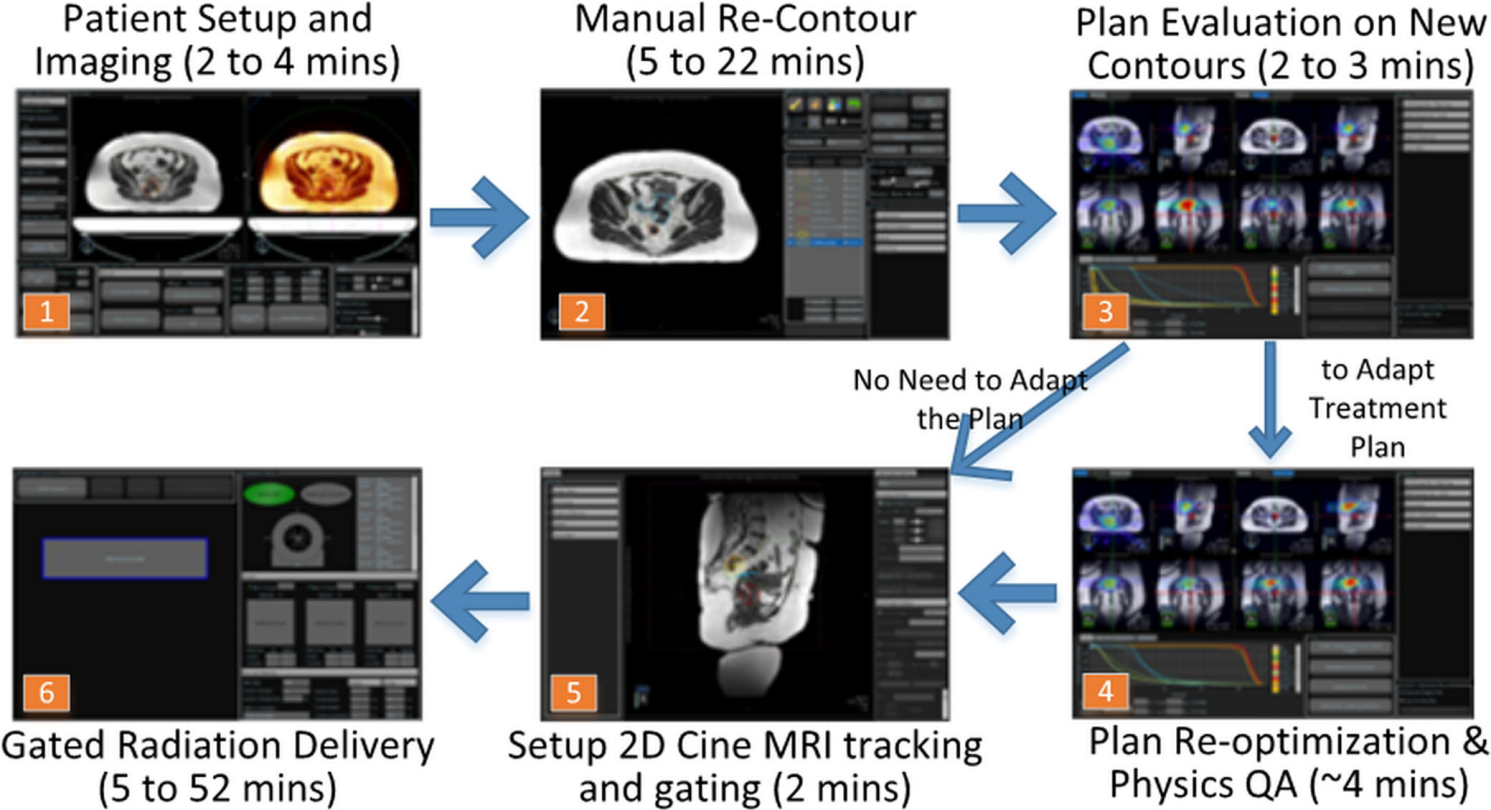
Workflow showing the steps and estimated timeline to the online adaptive MRgRT process.

The adaptive process workflow is shown visually in Fig. 1. To start the adaptive workflow, on each treatment day the most recently used treatment plan is loaded and a new volumetric MRI with exhale‐breath‐hold is scanned after patient setup. The couch table is shifted according to manual registration of the visible GTV in the new image with the GTV in the loaded plan. Following this registration, all other contours in the loaded plan are then rigidly copied onto the new images. All OARs within 30 mm of the PTV are re‐contoured on the new MR image by the attending physician. The overwhelming majority (>90%) of plans in this study had no GTV contour change. If the GTV is recontoured, most commonly at a patient’s first adapted treatment fraction, the PTV is regenerated by expanding the GTV by an isotropic 5 mm margin. The attending medical physicist reviews the manual OAR segmentation and assesses for other organ changes such as known areas of heterogeneity and skin surface position. Note that other institutions may choose to use the original simulation plan as the starting point for all online adaptive plans.^15^ The use of the most recently treated plan for this purpose is simply the chosen workflow at our institution.

The new radiation dose distribution is then calculated by applying the loaded plan to the current day’s anatomy. Target coverage and OAR doses are reassessed relative to plan goals and constraints. Only if target coverage can clearly be improved or any critical OAR dose constraint is not met will a new plan be created. The new plan is developed by reoptimization using the same beam angles and objective functions established in the currently loaded plan. In rare cases (~4%), attempts are made to adjust optimization objectives to further reoptimize the plan. After the plan is reoptimized, the attending physician evaluates and approves the plan. Physics quality assurance steps are taken next,^42^ and then the plan is delivered at free breathing, with real‐time, continuous 2D cine MR guidance for gating at the planned end‐of‐exhalation phase.^41^

All plans discussed in this work were developed with *OAR isotoxicity prioritized over target coverage*. Essentially, the OAR_CRIT_ constraints discussed above were hard constraints that must not be exceeded, regardless of target coverage. Therefore the goal of the model predictions in this work is identifying improvements to GTV coverage while maintaining the same OAR avoidance. By contrast, most previous KB prediction models aimed at identifying planning improvements related to OAR dose sparing while maintaining uniform target coverage, eg PTV V95% > 95%.^23^, ^24^, ^26^, ^27^, ^31^

### 2.C GTV 3D Dose Distribution Prediction ANN Model

In previously published KB methods, parameters associated with target size/shape, distance relationships between the target and OARs, and patient size were deemed important for predicting OAR 3D dose, with the most critical parameter the minimum distance from a voxel inside an OAR to the target surface.^23^, ^26^, ^27^ We have a similar approach in this study to predict 3D dose distributions inside the GTV. Our model input parameters include the minimum distances from a voxel inside the GTV to surfaces of various OAR structures (OAR_CRIT_, OAR_5mm_, etc.). To improve the prediction model accuracy and robustness, additional parameters derived from the distance and geometrical relationships between OARs and the GTV were added and are explained in Table 3. GTV V95 and D95 were found to strongly correlate with these additional parameters. It is important to note that we did not include any beam parameters (beam angles or fluence) so that the developed models could predict 3D dose before a treatment beam plan is devised. This is different from some previous work using ANN models to make 3D dose predictions.^26^, ^27^

**Table 3:** List of 16 ANN model input variables.

#	ANN Input Variables	#	ANN Input Variables
1	% of all voxels within 5 mm occupied by OAR_5 mm_	9	Square root of min distance to GTV surface
2	% of all voxels within 10 mm occupied by OAR_5 mm_	10	Min distance to OAR_5 mm_ (>=0 only)^b^
3	% of all voxels within 15 mm occupied by OAR_5 mm_	11	Min distance to OAR_5mm_ (<=0 only)
4	% of all voxels within 20 mm occupied by OAR_5 mm_	12	Square root of min distance to OAR_5mm_
5	% of GTV occupied by OAR_5 mm_ ^a^	13	Min distance to PTV_OPT_ (>=0 only)
6	Distance to GTV centroid	14	Min distance to PTV_OPT_ (<=0 only)
7	Max distance from GTV centroid to GTV surface^a^	15	Min distance to (OAR_5 mm_‐OAR_CRIT_) (>=0 only)
8	Min distance to GTV surface	16	Min distance to (OAR_5 mm_‐OAR_CRIT_) (<=0 only)

Each variable was calculated for all voxels inside the GTV for each plan unless indicated otherwise. Some ANN model input variables could have both positive and negative values, with negative values corresponding to an overlap between the GTV and the OAR structure associated with the variable.

^a^ Every voxel for the GTV had the same value.

^b^ A linear correlation was observed between this distance metric and dose but only up to a distance of ~ 16 mm. So this metric has a maximum value of 16 mm for any voxel.

**Fig. 2:**
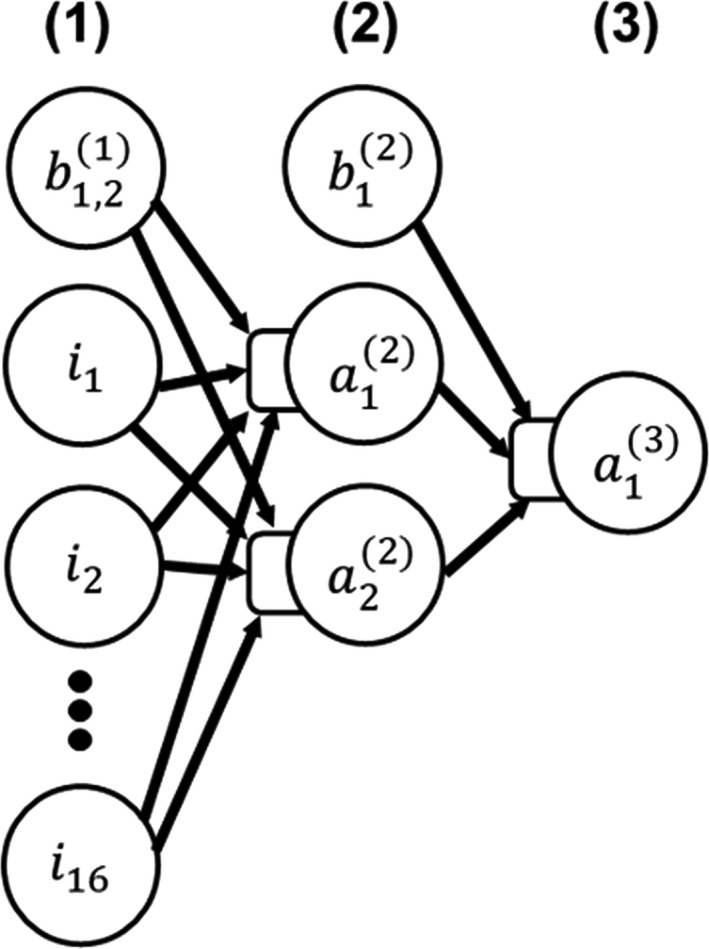
The ANN models used in this work, with three distinct layers: (1) input layer with 17 (16 inputs + 1 bias) nodes, (2) hidden layer with two activation nodes and one bias node, and (3) output layer with one activation node. The arrows represent the connections between nodes in each layer and are mathematically associated with the weighting parameters that get updated and optimized during model training.

In a separate study, we determined that for linac‐based MRgRT plans, plan quality comparisons between online adapted plans and fully optimized offline simulation plans showed no statistically significant differences. Therefore, we developed our models using both offline (simulation) and adapted plans. The model prediction output was per voxel dose value normalized to Rx. By tracking the model prediction accuracy in preliminary experiments, we identified the most relevant input variables, eliminated the less significant ones, and simplified the ANN models while avoiding overfitting. Our final ANN model was a two‐layer feed forward neural network design (one hidden layer: 2 nodes, one output layer: 1 node), shown in Fig. 2. It was implemented using MATLAB’s neural network toolbox. The default Levenberg‐Marquardt backpropagation was used for model training. Other models with more nodes in the hidden layer (eg up to ~ 5) provided very similar results if they were similarly optimized. Continuing to increase the number of nodes further led to model overfitting and clearly reduced model performance. Adding more hidden layers was similar. As a result, we chose to keep the simplest model possible that provided equal performance which was a model with two nodes in a single hidden layer. A wide range of different loss functions were also tested, including some that weighted different types of voxels differently.^26^ But the loss function that provided the most consistent prediction accuracy among the many that were tested was mean square error.

**Fig. 3:**
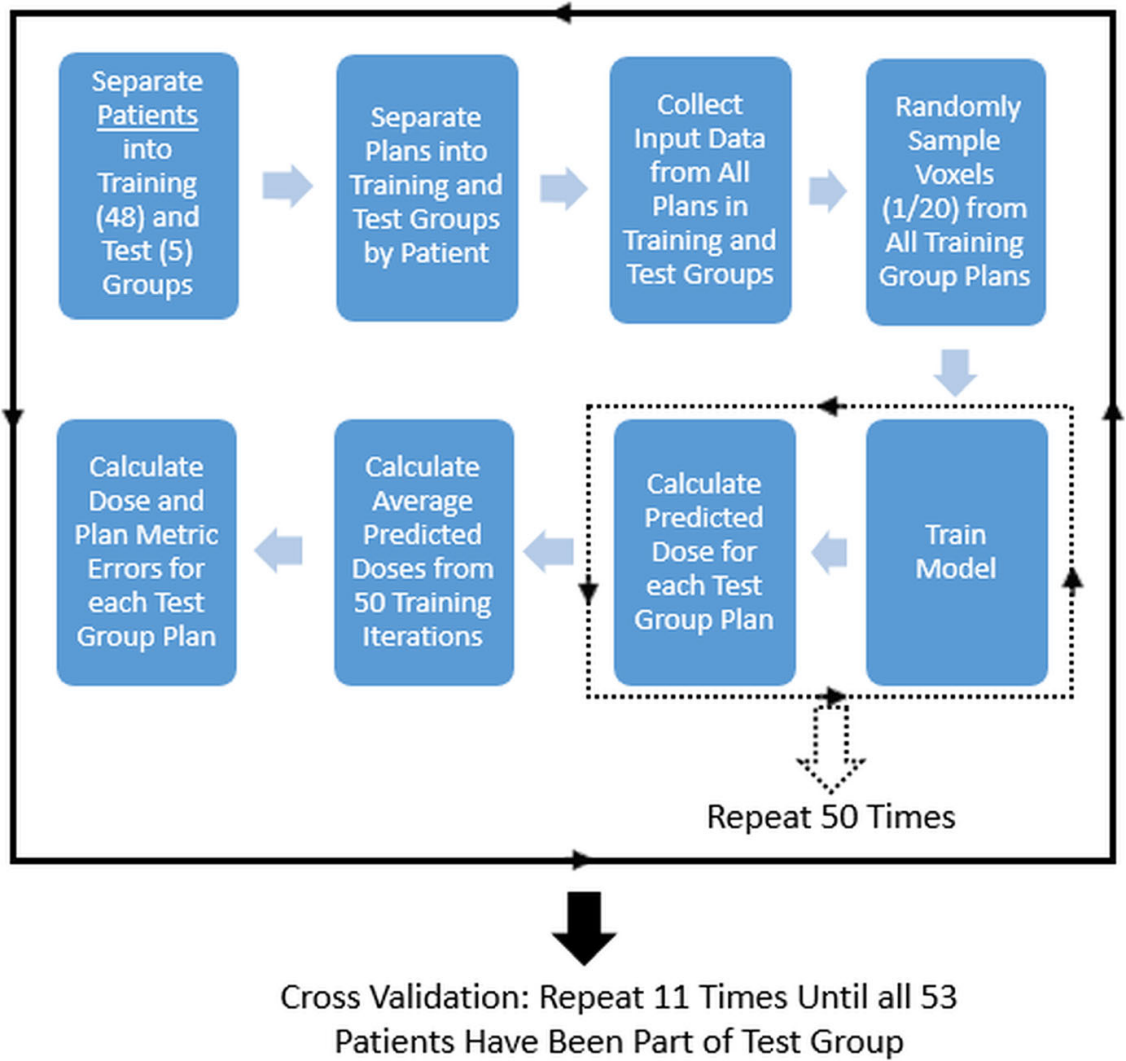
Process flowchart for ANN model training and testing

A flowchart for ANN model training, testing, and development is shown in Fig. 3. The simple ANN model design we eventually adopted allowed for very fast model training. So it was straightforward to utilize cross validation to train and validate the ANN models. We were not limited to training potential models and then testing on a small group of separate test patients/plans only once. The total 310 plans (50 simulation, 260 adapted) were separated into training and testing groups as follows: the 53 patients were split into ten groups of five and one group of three, then each group was cycled through as the test group. For each iteration of the cross validation, all of the plans from the test group patients made up the testing data while all the plans from the other ten groups served as training data. The cross validation was done on a patient‐by‐patient basis instead of a plan‐by‐plan basis to avoid testing the models on any plan coming from a patient whose plans were already used for model training.

The ANN model datasets in each cross validation iteration were quite large, with the typical training group composed of ~250 plans from 48 patients. The total number of voxels available in such a training group dataset was >2 million. It was not necessary to use all available voxels in any given cross validation to produce optimized and consistent model performance. So GTV voxels for each plan from the training group were randomly sampled at a 1/20 ratio and then accumulated to make the training dataset. This data sampling was chosen in an effort to fairly sample each set of GTV voxels regardless of GTV size differences in the group while achieving reduced model training time and consistent prediction results. Each cross validation iteration utilized ~120,000 voxels after data sampling.

Once the training dataset from randomly sampled GTV voxels was created, it was further separated into “training” and “validation” subsets at 85% and 15% respectively. The model was trained with the “training” dataset until six consecutive error increases were observed on the “validation” dataset or a maximum of 200 epochs was reached. Many different epoch cut‐offs were also tested, but the results did not strongly depend on this variable. The final training method was chosen based on allowing the model to either naturally find its optimal number of epochs based on error increases in the “validation” dataset, or run through a sufficient number of epochs to reach model convergence.

In an effort to ensure consistent model prediction performance, the model training sequence was repeated 50 times and the predicted dose results on each plan in the test group were averaged. The mean, mean absolute, and standard deviation errors of each entire 3D dose distribution in the test group were calculated, as well as mean dose (D_mean_), V95, V100, and D95. DVHs for each real and predicted dose distribution and their differences were determined (% GTV volume error as a function of dose). This process was repeated for each iteration of the cross validation until all patients were part of the test group, and then the overall results from the cross validation were accumulated.

### 2.D Outlier Detection, Rejection, and Model Refinement

One main application of the prediction models is to identify inferior plans by comparing the predicted plan quality metrics to those computed in the actual plans. This concept also allows model refinement by excluding inferior plans from training.^25^, ^26^ To do so, inferior plans were identified as any with an observed plan metric (V95, V100, or D95) outside the 95% prediction interval (PI) of the original ANN model, with:

95%PI=meanerror+1.96∗standarddeviationoferror

The inferior plans outside the 95% PI were then discarded and the model was retrained. After two rounds, a total of 25 inferior plans were identified and discarded. The model errors and results are included only for the refined model with 285 plans.

## 3 RESULTS

The prediction dose error statistics from the cross validation analysis are shown in Table 4. Dose prediction error was computed as *ΔDose = D_actual_* – *D_predicted_*, then averaged across all plans of all patients. Many different combinations of input variables could produce similar prediction dose errors yet distinct plan metric prediction errors. So it was important to track plan metric prediction errors during model optimization as well. In the end the most optimized ANN model was chosen based on the best balance between dose and plan metric error. As shown in Table 5, overall plan metric prediction errors for V95, V100, D95, and D_mean_ were generally low with mean absolute errors of 1.1%, 1.5%, 1.9 Gy, and 2.2 Gy respectively. Removing inferior plans to make the refined model helped to enhance its predictive ability for dose values relevant to plan quality, despite only marginal improvements in overall dose prediction errors.

**Table 4:** ANN model 3D dose prediction results.

Dose Prediction Error Mean ± σ (% Rx)	Absolute Dose Prediction Error Mean ± σ (% Rx)
0.1 ± 3.4 Gy (0.1 ± 6.2%)	3.5 ± 2.4 Gy (6.4 ± 4.3%)

The listed errors represent the averages on a plan‐by‐plan basis.

**Table 5:** ANN model plan metric prediction results.

**Metric**	Prediction Error	Absolute Prediction Error	95%	R^2^
V95 (%)	−0.1 ± 1.5	1.1 ± 1.1	3.3	0.97
V100 (%)	−0.5 ± 2.1	1.5 ± 1.5	5.0	0.95
D95 (Gy)	−0.9 ± 2.2	1.9 ± 1.4	4.6	0.95
D_mean_ (Gy)	0.1 ± 2.7	2.2 ± 1.6	5.1	0.91

Averages across all plans for both raw and absolute prediction errors are given. 95%: the value required to encompass the absolute error from 95% of all plans in the model. R^2^: coefficient of determination values for the linear fit of actual vs predicted plan metric values.

The dose prediction errors observed here are similar to previous efforts at 3D dose predictions using ANN models,^26^, ^27^ where errors ranged from 2‐10% of Rx generally but had some contributions up to 20% and greater. Care must be taken to recall that the fair comparison to previous results with this work would be in relating published OAR dose errors to GTV dose errors here. Previously published models focusing on OAR dose distributions also made predictions for GTV/PTV dose,^26^, ^27^ but due to the key differences in treatment planning strategy and design discussed in Methods, a direct comparison to previous GTV/PTV dose prediction results is not fair. Furthermore, the doses being predicted in this work are high, with the majority over Rx. As a result, the raw and % of Rx errors will appear to be higher than models focusing on OAR dose predictions.

**Fig. 4:**
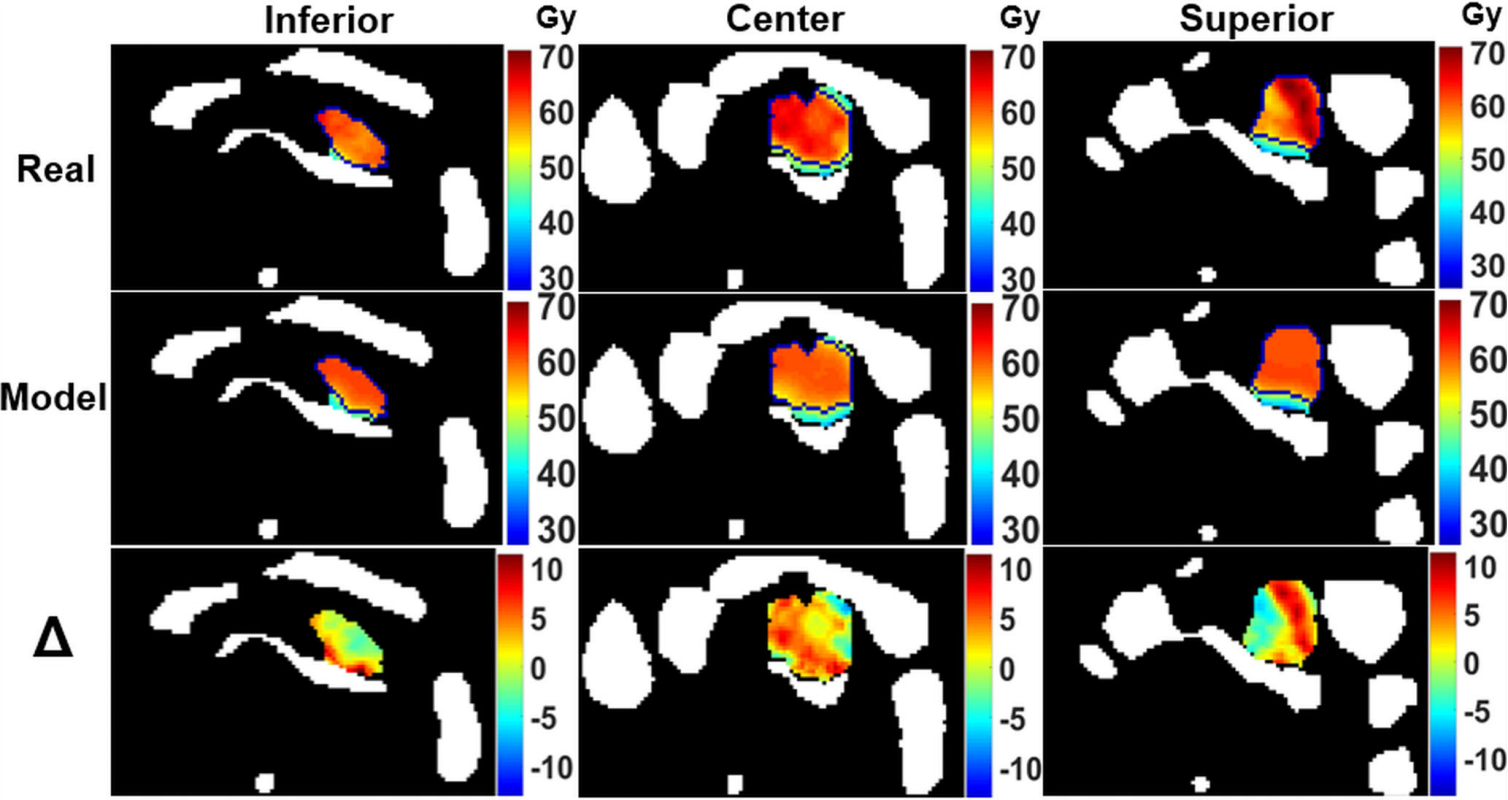
Three axial slices (inferior, center, and superior of GTV) of planned dose, model predicted dose, and *ΔDose* of a representative patient plan (SBRT: R_x_ = 50 Gy, OAR_CRIT_ constraint = 36 Gy). All OARs (stomach, duodenum, small bowel, large bowel, and spinal cord) are shown in white. The prescription isodose lines are shown in blue for the planned and predicted dose views. Dose prediction errors = 0.7 ± 3.9 Gy, absolute errors = 3.3 + 2.3 Gy. The plan metrics (planned/predicted) are V95 = 90.4%/88.5%, V100 = 86.8%/85.1%, D95 = 42.6 Gy/41.4 Gy, D_mean_ = 57.2 Gy/56.6 Gy.

Three axial slices of the planned dose, predicted dose, and the prediction error for a representative pancreas SBRT plan are shown in Fig. 4. It is evident that the predicted dose distribution is smoother than the planned dose because the prediction models did not consider beam angles. As a result, the dose distribution heterogeneity and hot spots are not easily predicted. Nevertheless, the prescription isodose lines are very similar between the planned and predicted doses, suggesting that the model can accurately predict dose values near Rx as well as clinical DVH metrics. Similar effects were observed generally throughout the model predictions. Note that the example in Fig. 4 was chosen as representative of typical predictions but not necessarily matched with all average metrics tracked.

**Fig. 5:**
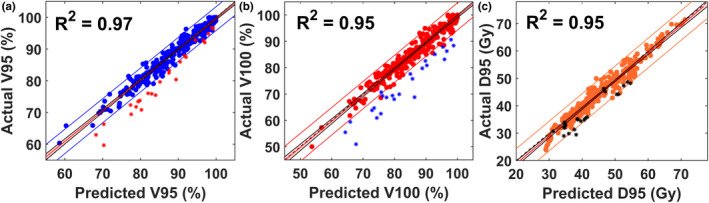
Results for (a) V95, (b) V100, and (c) D95 predictions calculated from the model‘s 3D dose predictions. The 45° dashed lines in each plot represent where the predicted and actual values are equal. The linear fit line and coefficient of determination (R^2^) are included. The outside boundary lines represent the 95% PI. The 25 inferior plans identified during model refinement are shown as *.

**Fig. 6:**
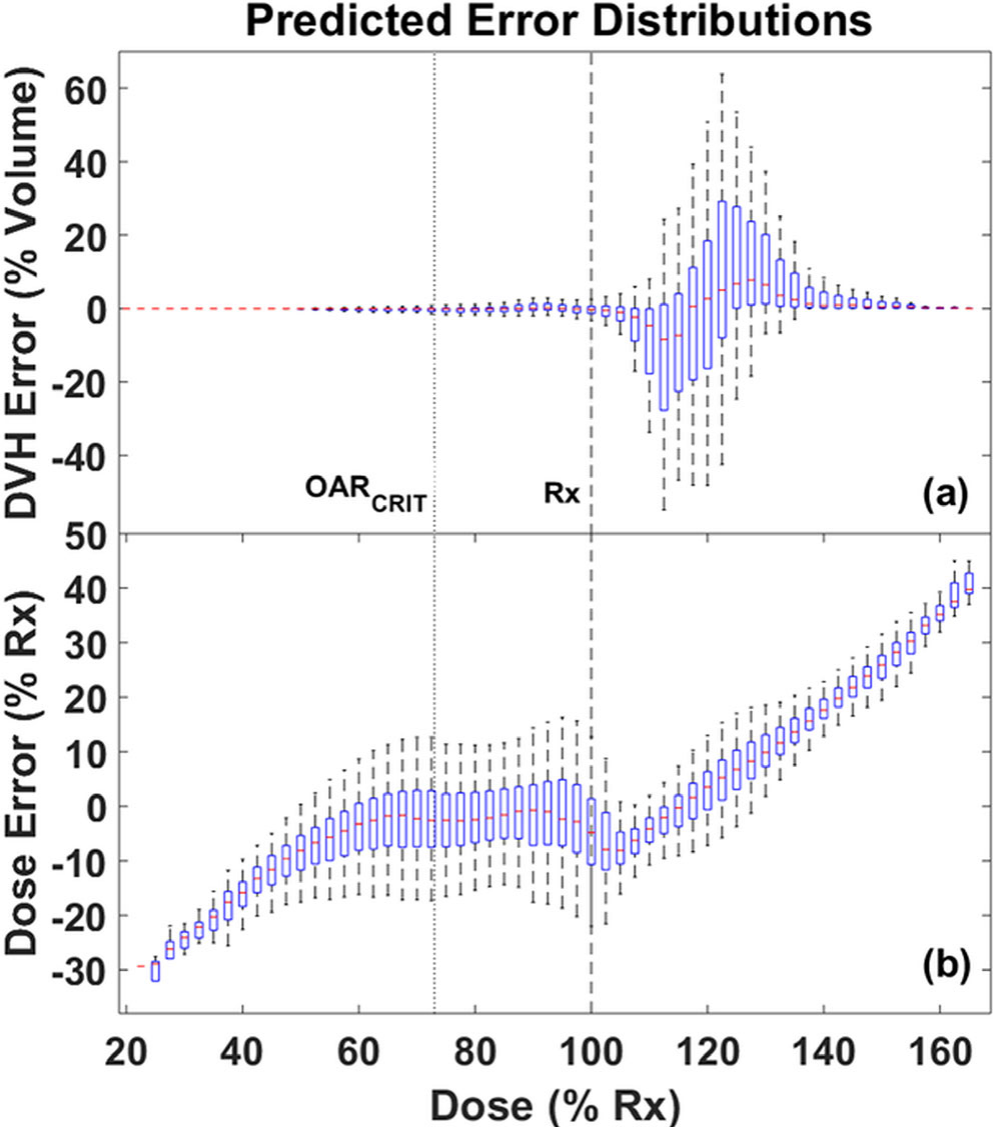
Distributions of prediction error for (a) Vx: the percentage of the GTV volume receiving dose of at least x percent of Rx (i.e. V20 through V160), and (b) dose in % Rx. The error distributions are shown as boxplots with a horizontal red line for the median, blue box encompassing the 25% and 75% interquartile ranges, and dotted line extending to the 95% range. The OAR_CRIT_ constraint (dotted) and Rx (dashed) levels are shown in the figures.

The results of some predicted plan metrics vs their true plan values are shown in Fig. 5. Overall, V95, V100, and D95 are well‐predicted, with R^2^ values of 0.97, 0.95, and 0.95, respectively, and linear fit slopes all near unity. The 25 inferior plans were well‐identified by their divergence from the 95% PI for the V95 and V100 plan metrics. Figure 6(a) shows results for comparisons between the real and predicted DVH metrics: error in Vx as a function of x where x is the percentage of Rx (eg V95 = % of GTV volume receiving 95% of Rx). It is clear that Vx is predicted well for x < 100% and large errors only occur for x > 100%. Figure 6(b) presents the overall dose prediction error distributions in % Rx also as a function of x, confirming that per voxel dose values are predicted well from x = 60%–100%.

## 4 DISCUSSION

ANN models were designed in this study to predict 3D dose distributions based on the average of prior plans used for model training. Our models allow a direct, 3D dose comparison between the history of previously treated plans and upcoming plans for future patients without needing to take the time and effort to create an actual treatment plan. This is possible because our models are based on inputs that require only target and OAR structure data, not planned beam parameters. Because beam angles and related information were not included as input variables, the models generally do not predict beam geometry‐dependent dose distribution heterogeneity and hot spots for each individual patient’s plan. While overall raw dose errors were not ideally low, the larger errors were generally associated with localized dose heterogeneity and high dose regions above Rx (Fig. 6). From a clinical standpoint, such errors are less relevant and do not affect predictions for important DVH metrics such as V95, or regions of the GTV receiving dose below Rx due to OAR sparing and constraint requirements. Such overall plan metrics and the predicted balance between OAR sparing and GTV coverage are the most important to guide adaptive decision‐making.

Due to the time constraints associated with the online adaptive process, adapted plans are not afforded the same level of optimization and scrutiny as high‐quality offline plans. The 3D dose prediction models establish benchmarking that can enable a rapid evaluation of online adaptive plans in terms of both plan quality and the overall dose distribution. Examples that showcase this idea are evident in Fig. 5. The 25 plans deemed inferior during model refinement are distinct from the general model predictions, with predicted values outside the 95% PI of at least one plan quality metric. Future treatment plans that are inferior to the history of previously treated plans could likely be identified similarly by testing with the model.

Several of the 25 plans identified as inferior were retrospectively replanned to assess for potential improvement. Conditions that would be present during real‐time adaptation were simulated for the replanning: no OAR constraints could be exceeded, optimization parameters could be manually tweaked and adjusted, and time constraints for the reoptimization process were kept in mind (~5 min maximum time allowed). Table 6 shows the results of the replanning analysis. While the degree of improvement varied across the plans tested, all plans that were reoptimized are closer to the quality claimed as possible by the ANN prediction model. Generally, from 40–100% of the difference between the predicted plan metrics and the original values were recovered after replanning. These results help to further showcase the clinical relevance and utility of the dose prediction models.

**Table 6:** Results of the replanning by manual reoptimization for several plans from the group of 25 identified as inferior by the ANN prediction models.

Metric	Type	Plan 1	Plan 2	Plan 3	Plan 4
V95 (%)	Clinical	52.6	82.3	72.6	80.4
	RePlan	63.3	87.9	76.3	83.1
	ANN	61.9	91.5	81.0	86.0
V100 (%)	Clinical	45.6	70.9	65.0	70.7
	RePlan	60.0	84.5	70.4	75.6
	ANN	60.7	89.0	79.3	82.4
D95 (Gy)	Clinical	23.0	36.6	29.3	35.6
	RePlan	22.7	38.9	35.2	37.3
	ANN	23.5	42.3	38.0	39.1

Despite the potential applications of the models developed in this work, there are limitations that must be addressed. First, the models were developed using only patient and plan data from a single institution. Additionally, the inability of the models to account for hotspots or other dramatic dose distribution changes prevents clinical applications that relate directly to hotspots.^43^, ^44^ Dose prediction models would need to include more detailed plan information related to beam angles and/or fluence^26^, ^27^ in order to more accurately predict such metrics.

In future applications, the predicted 3D dose distributions could serve as an alternative input to the treatment plan development process.^26^, ^30^ This is a particularly desirable strategy for online adaptive plans, much in the same vein as it is being pursued for automated treatment planning. An estimated 3D dose prediction tailored to the specific anatomy of the day could provide a much improved starting point for subsequent adapted plan development and optimization each fraction. The fact that our ANN models can provide a 3D dose prediction using contour information alone (a fully developed treatment plan is not needed) helps to bolster their potential use as novel inputs for alternative treatment planning strategies.

Additional future work that could extend this study relates mostly to improving the 3D dose prediction models. ANN model development and performance are based mainly on the ability to identify key input data that strongly relates to making accurate output predictions. Most likely, the best 3D dose prediction models would be based on convolutional neural networks (CNNs),^29^, ^30^, ^31^ which have shown improved dose prediction performance relative to what was achieved in this study. However, CNN models are well known to require vast amounts of training data to learn the necessary relationships to make accurate and robust predictions.^29^, ^30^, ^31^ As the number of patients treated with online adaptive MRgRT continues to increase, future work may be possible related to CNN based prediction models with improved capabilities.

## 5 CONCLUSION

We developed ANN models to predict 3D dose distributions and overall plan quality metrics for abdomen cancers in adaptive MRgRT. The prediction models will be useful to improve adaptive planning strategies and workflows through more informed plan optimization and evaluation in real time.

## CONFLICT OF INTEREST

The authors have no conflicts to disclose.
